# Synthesis, Optimization, and Evaluation of a New Sustained-Release Food Formulation for *Polygonatum sibiricum* Polysaccharide

**DOI:** 10.3390/foods15010147

**Published:** 2026-01-02

**Authors:** Wenjie Qu, Zhuoyuan Zhang, Yiran Guo, Yan Chen, Linpeng Wang, Jian Huang, Jiyong Yin

**Affiliations:** Key Laboratory of Public Nutrition and Health, National Health Commission of the People’s Republic of China, National Institute for Nutrition and Health, Chinese Center for Disease Control and Prevention, Beijing 100050, China; qwj370612@163.com (W.Q.); zhangzy@ninh.chinacdc.cn (Z.Z.); guoyr@ninh.chinacdc.cn (Y.G.); chenyan7341@163.com (Y.C.); 18596257957@163.com (L.W.); huangjian@ninh.chinacdc.cn (J.H.)

**Keywords:** *Polygonatum sibiricum* polysaccharide (PsP), hydroxyapatite (HAP), sustained release, response surface method (RSM), evaluation

## Abstract

*Polygonatum sibiricum* polysaccharide (PsP), one of the main components of *Polygonatum* *sibiricum* used in traditional Chinese food and medicine, has important bioactive functions, but it is difficult to fully utilize PsP because of the degradative effect of digestive gastric juices. This study aimed to innovatively synthesize a new food formulation for PsP, namely, a PsP–hydroxyapatite (HAP) sustained-release system, so as to reduce its degradation. The new food formulation was optimized and evaluated by the response surface method (RSM) and by in vitro experiments. The optimal stirrer temperature, reaction pH, etching pH, and loading time for synthesizing PsP-HAP were 85.62 °C, pH 11.12, pH 8.40, and 5.10 h, respectively, all of which were different from the findings of other similar research studies. The average encapsulation rate of PsP-HAP reached (40.16 ± 1.54)%, and the content of PsP was 8.98%. Additionally, PsP-HAP appeared to be pH-responsive, and its continuous antioxidative effect was first proven by the DPPH assay and then cytologically by a total antioxidative capacity assay. The CCK-8 assay indicated that PSP-HAP did not induce toxicity. This study successfully developed a new food formulation for PsP which appears to have the potential to reduce the degradative effect of digestive gastric juices. Thus, it is possible to achieve full utilization of PsP by using this new sustained-release food formulation.

## 1. Introduction

*Polygonatum sibiricum* is one of 110 kinds of traditional Chinese food and medicine and has a series of healthy effects, including enhancing immunity, antioxidative effects, and anti-fatigue effects. *Polygonatum sibiricum* polysaccharide (PsP), the main active component of *Polygonatum sibiricum*, is a high-molecular-weight compound composed of approximately 25 monosaccharides linked by glycosidic bonds [1]. Many research studies have shown that PsP has multiple bioactive functions, which include regulating lipid metabolism [2,3], enhancing immunity [4], reducing blood glucose [5], anti-inflammatory effects [6], and antitumor effects [7,8].

Our team previously conducted a series of research studies exploring extraction techniques for PsP and its functional effects. We first used the ultrasound-assisted extraction technique with deep eutectic solvents (UAE-DES) to extract PsP [1], which not only increased the extraction yield (43.61%) but also kept the structural stability and antioxidative ability of PsP. Then, we confirmed that a higher extraction yield of PsP could be achieved from mature rhizomes, whose PsP had a higher total antioxidative capacity than that from young rhizomes, while the PsP from young rhizomes had a higher DPPH radical scavenging capacity [9]. Our research also indicated that PsP that was extracted by the UAE-DES technique possessed a higher antioxidative capacity compared with other extraction techniques, including water–ethanol extraction, ultrasound extraction, and deep eutectic solvent extraction [10]. After that, a cell model of lipid ectopic deposition, which was induced by palmitic acid (PA), was employed by our team to verify the preventive effect of PsP on lipid ectopic deposition. The results not only indicated that PsP can alleviate oxidative stress [11] and lipid ectopic deposition in the cell model but also identified the role of PsP in the AMPK/PPARγ/PGC-1α signaling pathway as part of the mechanism preventing lipid ectopic deposition. These findings suggested that PsP might have the potential to prevent sarcopenic obesity.

As the initial plan, we wanted to further verify the results obtained from the cell experiments in animal experiments using gavage. However, our preliminary experiment indicated that PsP could not be fully utilized in animal experiments due to the degradative effect of the gastrointestinal tract [12] on it. Therefore, we need to use a new kind of food formulation with a sustained-release effect to continuously release PsP so as to reduce the degradation of PsP and to stably maintain its content, thus achieving full utilization of PsP.

Food formulations with a sustained-release effect generally adopt physical adsorption and chemical interactions such as hydrogen bonding and van der Waals force [13] to achieve incorporation of the carrier and the substance to be delivered. Then, the incorporated system gradually releases the substance to be delivered into the physiological environment according to their different characteristics; this can include physical controlled release, diffusion, etc., which ensures stable delivery of the substance into the body and thus the ability to fully utilize the delivered substance.

Currently, the carriers that can realize a sustained-release effect include hydroxyapatite (HAP), liposomes, poly(lactic-co-glycolic acid) (PLGA), nanomicelles, and exosomes. Among these, HAP has excellent biocompatibility and biosafety, and it does not affect hemolysis; platelet adhesion, aggregation, and activation; or the coagulation system [14]. In addition, it is easy to control its self-morphology. By adjusting the synthetic process or parameters, HAP with different particle sizes, morphologies, and porosities can be synthesized, which is not only able to be used to meet the requirements of different substances to be delivered for a sustained-release effect but can also avoid burst release. The above-described HAP performances have appeared in many research studies on traditional Chinese food and medicine, bioactive components, and medicine; for example, one study about the bioactive components of traditional Chinese food and medicine indicated that the combination of HAP and curcumin could sustainably release curcumin over 14 days [15], which is clearly longer than the release time of nanostructured lipid carriers loaded with curcumin [16]. One study on medicines also showed that a system incorporating amoxicillin and nano-HAP can achieve the sustained release of amoxicillin over 30 days [17], which is much longer than the sustained-release effect (only 22 h) of polymeric nanoparticles loaded with amoxicillin [18]. Therefore, HAP has been widely used in combination with natural phytochemicals, antibiotics, growth factors, and antitumor drugs in food, medicine, and other fields that require a sustained-release effect.

However, there is no investigation into loading PsP into HAP so as to sustainably release PsP, at present. Can we adopt HAP to carry PsP to reduce the degradation of PsP and to stably maintain its content, thus achieving fully utilization of PsP?

The objective of this research study was to synthesize a new food formulation of PsP which can sustainably release PSP so as to keep the structural stability of PsP, to reduce its degradation, and to fully utilize it. In order to realize this, we adopted HAP to load PsP (PsP-HAP) by the CaCO_3_ template method. Then, the optimal parameters of the PsP-HAP system were determined by using the RSM. After that, the sustained-PsP-release effect of PsP-HAP was determined by using sustained-release and antioxidative experiments. We aimed to obtain a new food formulation of PsP, the PsP-HAP sustained-release system, that possesses excellent PsP-loading ability and sustained-PsP-release performance so as to achieve full utilization of PsP in functional food.

## 2. Materials and Methods

### 2.1. Materials

The C2C12 skeletal muscle cell line was purchased from the Cell Resource Center, Institute of Basic Medical Sciences (Beijing, China). All chemical reagents and materials used in this research study are listed in Table 1.

### 2.2. Main Instruments and Equipment

The following were used: magnetic stirrers (IKA, Inc., Staufen, Germany) [10,11]; peristaltic pump (Shanghai Yanyao Technology Co., Ltd., Shanghai, China); circulating water multi-purpose vacuum pump (Gongyi Yuhua Instrument Co., Ltd., Zhengzhou, China) [10,11]; U-3900 spectrophotometer (Hitachi, Ltd., Tokyo, Japan); Allegra x-22 R centrifuge (Beckman coulter, Inc., Brea, CA, USA) [10,11]; SpectraMax I3X Enzyme marker (Molecular Devices Instruments Ltd., San Jose, CA, USA) [10,11]; electric heating blast drying oven (Shanghai Yiheng Scientific Instrument Co., Ltd., Shanghai, China) [10,11]; muffle furnace (Thermo Fisher Scientific Inc. Waltham, Massachusetts, USA); MCO-18AIC CO_2_ Incubator (Panasonic Corporation Co., Ltd., Tokyo, Japan) [10,11]; Milli-Q^TM^ Reference Ultrapure Water Purification System (Sigma Co., Ltd. Louis, MO, USA) [10,11]; JSM-7500F Field-Emission Scanning Electron Microscope (Jeol Ltd., Akishima, Tokyo, Japan); Mastersizer 3000 Laser diffraction particle size analyzers (Malvern Panalytical Ltd., Malvern, Worcestershire, UK); AutoPore V Mercury intrusion porosimeters (Micromeritics Instrument Corporation, Norcross, GA, USA); SDT Q600 simultaneous thermal analyzers (TA Instruments, LLC., New Castle, DE, USA).

### 2.3. Synthetic Process of PsP-HAP

The template method was adopted to synthesize PsP-HAP in this study. CaCO_3_ was added to 300.00 mL of deionized water after it was weighted accurately. Then, the suspension was heated in a water bath, and its pH was adjusted by using 5.00 M sodium hydroxide solution. At the same time, Na_2_HPO_4_ was weighted and was dissolved in 200.00 mL of deionized water, and the solution was heated in a water bath. After that, the Na_2_HPO_4_ solution was added to the CaCO_3_ suspension by a peristaltic pump at a rate of 2.00 mL/min. The solution pH was adjusted again, and the solution continued to react for 2.00 h after the addition was completed. The above process needed heating and stirring (50.00 °C, 500.00 rpm). After 2.00 h, HAP was collected by using suction filtration. Subsequently, HAP was acid-etched in a citric acid solution (0.10 mol) for 48.00 h, which removed residual CaCO_3_ and reshaped the surface of HAP.

Then, HAP and PsP were simultaneously added to 100.00 mL of deionized water, and the weight ratio of HAP and PsP was 1 to 2. PsP was loaded into HAP by continuously stirring the solution of PsP and HAP. Finally, synthetic PsP-HAP was collected by using suction filtration, and the moisture of PsP-HAP was removed by drying at 60.00 °C.

### 2.4. Single-Factor Experiments

Reaction pH (9.90, 10.60, 11.30, 12.00, and 12.70) [19], stirrer temperature (50.00, 60.00, 70.00, 80.00, 90.00, 100.00, and 110.00 °C) [20], CaCO_3_ dosage (1.50, 1.75, 2.00, 2.25, and 2.50 g) [21], etching pH (5.00, 6.00, 7.00, 9.00, 11.00, and 13.00), and PsP-loading time (0.00, 3.00, 6.00, 9.00, and 12.00 h) were used to conduct single-factor experiments.

In each single-factor experiment, only one factor was changed, and the other factors were fixed. The fixed values of reaction pH, stirrer temperature, CaCO_3_ dosage, etching pH, and PsP-loading time were 11.30, 50.00 °C, 2.00 g, 6.00, and 6.00 h, respectively.

### 2.5. Response Surface Method (RSM) [1]

The principle of Box–Behnken design was adopted to optimize the synthetic parameters of PsP-HAP [1]. Based on the results of the above single-factor experiments [1], four variables, i.e., stirrer temperature (A), reaction pH (B), etching pH (C), and PsP-loading time (D), were selected as factors of the response surface method. The encapsulation rate was used as the evaluation index.

The design factors and levels can be found in Table 2.

### 2.6. Physicochemical Characterization of HAP

The morphology of HAP was observed by a scanning electron microscope (SEM) after the sample was prepared by casting a drop of prepared material on a silicon wafer. The particle size of HAP was determined by a laser particle size analyzer (LDPSA) after the sample was ultrasonically dispersed in water for 15 min. The porosity was detected by mercury injection apparatus, and the porosity can be calculated using Formula (1):

(1) $$Porosity\left(\%\right)=\frac{V_{i}}{V_{s}}\times 100$$

V_i_: the total intrusion volume of mercury; V_s_: the volume of the sample.

Thermogravimetric analysis (TGA) was performed to evaluate the thermal stability of HAP. The temperature range was between room temp and 800 °C, and the speed of temperature rise was 10 °C/min.

### 2.7. Determination of Encapsulation Rate and PsP-Loading Capacity

#### 2.7.1. Encapsulation Rate

The decrement method was used to confirm the encapsulation rate of PsP. After the suction filtration of synthetic PsP-HAP was completed, the filtrate was detected by the anthrone–sulfuric acid method. The difference between the total added polysaccharide and the polysaccharide in the filtrate is the content of PsP that has been loaded into HAP.

The encapsulation rate can be calculated using Formula (2):

(2) $$Encapsulation\;rate\left(\%\right)=\frac{M_{o}-M_{j}}{M_{o}}\times 100$$

M_o_: the weight of the total added polysaccharide; M_j_: the content of polysaccharide in the filtrate.

#### 2.7.2. Loading Capacity

PsP-HAP was burnt by using a muffle furnace. The lost weight was regarded as the weight of PsP [22]. The loading capacity can be calculated using Formula (3):

(3) $$Loading\;capacity\left(\%\right)=\frac{M_{b}-M_{a}}{M_{b}}\times 100$$

M_b_: the weight of PsP-HAP before burning it; M_a_: the weight after burning it.

### 2.8. Release Profile

The pH values of three PBS solutions were adjusted to 2.00, 3.00, and 7.00 so as to simulate three kinds of environments: succus gastricus, lysosomes, and general physiological situation, respectively. Then, three portions of 0.20 g of PsP-HAP were added to the above three portions of PBS solutions (10.00 mL) to release PsP at 37.00 °C. For the each of the above conditions, the sustained-release solutions were collected 1.00 h, 2.00 h, 3.00 h, 4.00 h, 5.00 h, 6.00 h, 12.00 h, 24.00 h, and 48.00 h [23] after PsP-HAP was added to the PBS solution. The collected sustained-release solutions were detected by using anthrone–sulfuric acid method (U-3900 spectrophotometer at 582 nm). In the above process, the new PBS solutions were added to the system to conduct the next release of PsP after the last sustained-release solutions were collected. The cumulative PsP release rate can be calculated using Formula (4) [1]:

(4) $$Cumulative\;release\;rate\;\left(\%\right)=\frac{V_{0}C_{0}+V\sum_{1}^{n-1}C_{i}}{M}\times 100$$

V_0_: the total volume of the solution; C_i_: the concentration of polysaccharide in the sustained-release solution obtained at time i; V: the volume of the solution extracted at each time; M: the weight of PsP in PsP-HAP; n: the number of samplings.

### 2.9. DPPH Radical Scavenging Rate of PsP-HAP Sustained-Release System

PBS at pH 2.00 was used to release PsP from PsP-HAP, so as to obtain a sustained-release solution of PsP-HAP. PBS at pH 2.00 was used to dissolve HAP, and the solution was used as the negative control. The range of pH values of collected “sustained-release solutions” and “HAP solutions” was between pH 5.00 and pH 6.00. Then, in order to ensure homogeneity and comparability of the conditions of antioxidative experiments, the pH values of the above two groups were adjusted to a uniform value (pH 5.00). After that, the PsP solution was adjusted to pH 5.00, and the PsP solution at pH 5.00 was used as the positive control, while PBS at pH 5.00 was used as the blank control. Finally, the DPPH radical scavenging rate of antioxidative activity tests was performed at pH 5.00.

The sustained-release solutions of PsP-HAP were collected by using the same method as that described in “Section 2.8. Release Profile”. The timepoints at which we collected the sustained-release solutions were the 1st, 2nd, 4th, 8th, and 16th hour. Then, the PsP concentrations in the sustained-release solutions of PsP-HAP at the 5 timepoints were measured by using the anthrone–sulfuric acid method. The DPPH radical scavenging experiment for all sustained-release solutions was conducted after that. A 0.50 mL sample was mixed with 3.00 mL of DPPH solution, and then the mixture was placed at room temperature in the dark for 30.00 min [1,24]. At the last, the absorbance was measured at the 517 nm wavelength. The DPPH radical scavenging rate can be calculated using Formula (5):

(5) $$DPPH\;radical\;scavenging\;rate\left(\%\right)=\left(1-\frac{A_{j}-A_{i}}{A_{o}}\right)\times 100$$

A_j_: the absorbance of the sample; A_i_: the absorbance of methyl alcohol and DPPH solution; A_o_: the absorbance of deionized water and methyl alcohol [1].

In order to more comprehensively assess the DPPH radical scavenging effect of the PsP-HAP sustained-release system, the comparisons of the above results included two dimensions. The first dimension was the comparison of the DPPH radical scavenging rate of each group among different timepoints, and the second dimension was the comparison of the rate of different groups at each timepoint.

### 2.10. Cytotoxicity Evaluation for PsP-HAP Sustained-Release System

The cytotoxicity of PsP-HAP and HAP was evaluated by measuring the cell viability of C2C12 cells [25]. A total of 2.00 g of PsP-HAP and 2.00 g of HAP were separately added to 10.00 mL of medium at 37.00 °C, and they were continuously immersed for 24.00 h to obtain supernatants [25], which were used as the evaluation samples. Dimethyl sulfoxide (DMSO) was used as the positive control, non-toxic polyethylene was used as the negative control, and medium was used as the blank control. C2C12 cells were continuously cultured for 24.00 h after the above study substances were added to the cell plate.

Then, CCK-8 solution was added to each well of the cell plate, and the cells were incubated for 30.00 min [11]. Finally, the absorbance was measured at the 450 nm wavelength. The cytotoxicity of PsP-HAP and HAP was described by using cell viability activity. Cell viability activity can be calculated using Formula (6) [11]:

(6) $$Cell\;viability\;activity\left(\%\right)=\left(\frac{A_{j}-A_{i}}{A_{o}-A_{i}}\right)\times 100$$

A_j_: the sample; A_i_: the medium but not the cell; A_o_: the medium.

### 2.11. Total Antioxidative Capacity of PsP-HAP Sustained-Release System

The antioxidative capacity of PsP-HAP in the cell experiment was evaluated by the Total Antioxidant Capacity Assay Kit. PBS at pH 2.00 was used to release PsP from PsP-HAP so as to obtain a sustained-release solution of PsP-HAP. PBS at pH 2.00 was used to dissolve HAP. Normal medium was used to separately dilute the sustained-release solutions of PsP-HAP and HAP solutions to 1:3000, so as to obtain the study media of the PsP-HAP group and the negative group, and the pH value of all of these solutions was 7.50, which is the same as the pH value of normal medium. In order to ensure homogeneity and comparability of the conditions of antioxidative experiments, normal medium was used to dissolve PsP, and the PsP solution was used as the positive control, while normal medium was used as the blank control. Finally, the cells’ total antioxidative capacity test was performed at pH 7.50.

The sustained-release solutions of PsP-HAP were collected by using the same method as that described in “Section 2.8. Release Profile”. The timepoints at which we collected the sustained-release solutions were the 1st, 4th, 8th, and 16th hour. Then, the PsP concentrations in the sustained-release solutions of PsP-HAP at the 5 timepoints were measured by using the anthrone–sulfuric acid method. C2C12 cells were cultured in culture dishes. After the four kinds of study media were added to the culture dishes, the cells were sequentially cultured for 6 h. After that, the cells of each group were collected and ultrasonically disrupted to exact antioxidative substances. The antioxidative substances in all groups were adjusted to the same level by using the bicinchoninic acid assay (BCA) method, and their antioxidative capacities were detected by using the Ferric reducing antioxidant power (FRAP) method. In the calculation, the antioxidative capacity of the blank control was defined as 100%, and the antioxidative capacities of the other groups were calculated as the relative ratios to that of the blank group.

In order to more comprehensively assess the total antioxidative effect of the PsP-HAP sustained-release system, the comparisons of the above results also included two dimensions. The first dimension was the comparison of the total antioxidative capacities of each group among different timepoints, and the second dimension was the comparison of the capacities of different groups at each timepoint.

### 2.12. Statistical Analysis

SPSS19.0 (IBM, Armonk, NY, USA) was used for statistical analyses, and Origin 2018 (Origin Lab Inc., Hampton, MA, USA) was used to plot graphs [1]. In this study, one-way analysis of variance was used for response surface result analysis and comparison of means among multiple groups [1]. Each experiment was repeated at least three times, and the results are presented as means ± standard deviations (SDs). The significant level was set to α = 0.05 [1].

## 3. Results

### 3.1. Single-Factor Experimental Results

**Figure 1:**
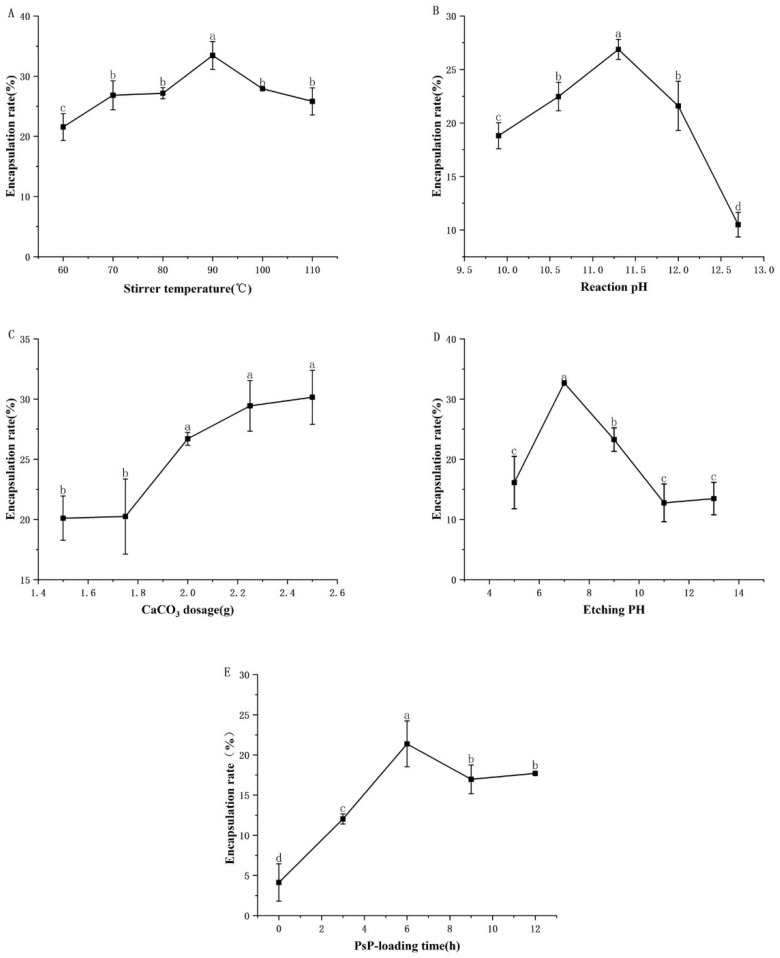
The results of single-factor experiments. Note: (**A**) Stirrer temperature. (**B**) Reaction pH. (**C**) CaCO_3_ dosage. (**D**) Etching pH. (**E**) PsP-loading time. Different lowercase letters indicate significant differences (*p* < 0.05).

The optimal results of the single-factor experiments on the conditions for synthesizing PsP-HAP are presented in Figure 1.

As shown in Figure 1A, the encapsulation rate increased gradually when the stirrer temperature ranged from 60.00 to 90.00 °C; then, it decreased gradually after 90.00 °C. The encapsulation rate of the stirrer temperature of 90.00 °C was significantly higher than that of the stirrer temperatures of 70.00 °C and 110.00 °C. In addition, all of them were significantly higher than that of the stirrer temperature of 60.00 °C. The differences in the above indicators were statistically significant (*F* = 12.794, *p* < 0.05). Therefore, the range from 60.00 to 110.00 °C was selected as the interval level of stirrer temperature in subsequent response surface experiments, and Table 1 is based on three specific temperatures.

As shown in Figure 1B, the encapsulation rate increased gradually when the reaction pH ranged from 9.90 to 11.30, while it decreased gradually at reaction pH beyond 11.30. The encapsulation rate of reaction pH 11.30 was significantly higher than that of reaction pH 10.60 and 12.00. In addition, all of them were significantly higher than that of pH 9.90 and 12.70. The differences in the above indicators were statistically significant (*F* = 60.975, *p* < 0.05). Therefore, the reaction pH range from 10.60 to 12.00 was selected as the interval level of reaction pH in subsequent response surface experiments, and Table 1 is based on three specific pH values.

As shown in Figure 1C, the encapsulation rate showed a continuously upward trend when the CaCO_3_ dosage was in the range from 1.50 to 2.50 g. After that, the purity of HAP appeared to decrease if the CaCO_3_ dosage was continuously increased. Therefore, the response surface experiments did not include CaCO_3_ dosage.

As shown in Figure 1D, the encapsulation rate increased gradually when the etching pH ranged from pH 5.00 to pH 7.00; then, it decreased gradually for etching pH beyond 7.00. The encapsulation rate of etching pH 7.00 was significantly higher than that of pH 9.00. All of them were significantly higher than that of pH 5.00. The differences in the above indicators were statistically significant (*F* = 41.070, *p* < 0.05). Therefore, the etching pH range from pH 5.00 to pH 9.00 was selected as the interval level of etching pH in subsequent response surface experiments, and Table 1 is based on three specific etching pH values.

As shown in Figure 1E, the encapsulation rate increased gradually when the PsP-loading time ranged from 0.00 to 6.00 h; then, it decreased gradually after 6.00 h. The encapsulation rate of the PsP-loading time of 6.00 h was significantly higher than that of the PsP-loading time of 9.00 h. All of them were significantly higher than that of the PsP-loading time of 3.00 h. The differences in the above indicators were statistically significant (*F* = 19.185, *p* < 0.05). Therefore, the PsP-loading time range from 3.00 to 9.00 h was selected as the interval level of PsP-loading time in subsequent response surface experiments, and Table 1 is based on three specific PsP-loading times.

### 3.2. Results of RSM Experiments

#### 3.2.1. Model Establishment and Data Fitting [1]

Table 3 presents the results of the RSM experiments [1], which were used to conduct fitting analysis, and the encapsulation rate can be calculated using Formula (7):

(7) $$\begin{array}{c} Encapsulation\;rate=38.73+0.2617\times A-1.54\times B+6.16\times C+0.6850\times D+\\ 1.19\times AB-0.8350\times AC-1.06\times AD-1.83\times BC+2.86\times BD\\ -0.2275\times CD-7.59\times A^{2}-5.70\times B^{2}-10.39\times C^{2}-6.82\times D^{2} \end{array}$$

A: the value of stirrer temperature; B: the value of reaction pH; C: the value of etching pH; D: the value of PsP-loading time.

The determination coefficient (*R*^2^) of this coded equation was 0.9726. The result of each experiment is shown in Supplementary Materials.

#### 3.2.2. Analysis of Variance (ANOVA)

Table 3 presents the ANOVA results. It shows that this model was significant (*F* = 35.49, *p* < 0.0001) and the lack of fit was not significant (*p* = 0.0830), indicating that the model had a good fitting effect and there was no obvious misfit factor. The coefficient of determination (*R*^2^) of the model was 0.9726, the adjusted coefficient of determination was 0.9452, the predicted coefficient of determination was 0.8517, and the signal-to-noise ratio was 19.5078. The above results indicate that the model can explain 85.17% of the variation in the encapsulation rate and can effectively analyze and predict the encapsulation rate. In terms of statistical significance, two linear terms (reaction pH and etching pH), one interaction term (reaction pH and PsP-loading time), and four quadratic terms of the model had significant effects on the encapsulation rate. Based on the *F*-values of each factor, the order of parameters according to their influence on the encapsulation rate appeared as follows: etching pH > reaction pH > PsP-loading time > stirrer temperature.

#### 3.2.3. Analysis of Contour Plots and 3D Plots

**Figure 2:**
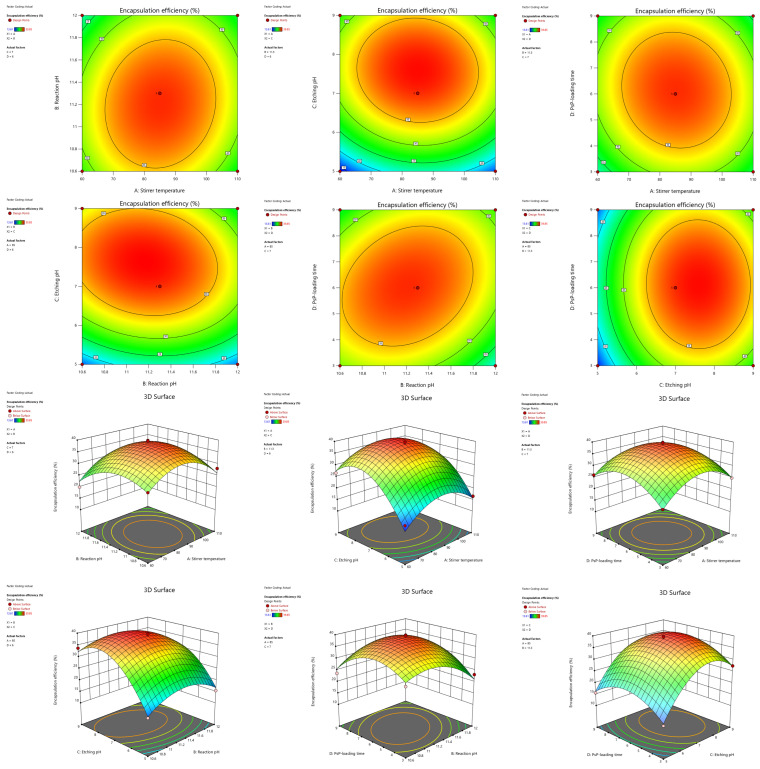
Contour plots and 3D plots of each interaction term. Note: Contour lines of elliptical shape indicate a significant interaction between two factors, while contour lines of circular shape indicate that there was no interaction [1]. The graphic color change from blue to red means that the encapsulation rate increased [1]. Faster color changes show a more significant effect of the factor on the encapsulation rate [1].

The interaction effects of various factors on the encapsulation rate can be determined through contour plots and 3D plots (Figure 2). It can be observed that the contour plots of BD (reaction pH and PsP-loading time) and BC (reaction pH and etching pH) appeared elliptical in shape, indicating there were interactive effects between the above two factor pairs. A comprehensive analysis indicated that the interactive effect of BD (reaction pH and PsP-loading time) was the strongest, an effect which was significant on the encapsulation rate (*F* = 10.09, *p* < 0.05).

According to the result of the ANOVA, contour plots, and 3D plots, the optimal process conditions were a stirrer temperature of 85.62 °C, a reaction pH of 11.12, an etching pH of 8.40, and a PsP-loading time of 5.10 h.

#### 3.2.4. Verification of Optimal Process Conditions

Three verification experiments under the optimal process conditions (stirrer temperature of 85.62 °C, reaction pH of 11.12, etching pH of 8.40, and PsP-loading time of 5.10 h) indicated that the average encapsulation rate was (40.16 ± 1.54)% and the error between the average value and the predicted value was 2.42%. These results determined that the model had good predictive ability, and the optimized process parameters were reliable [1].

### 3.3. Physicochemical Characterization of HAP

**Figure 3:**
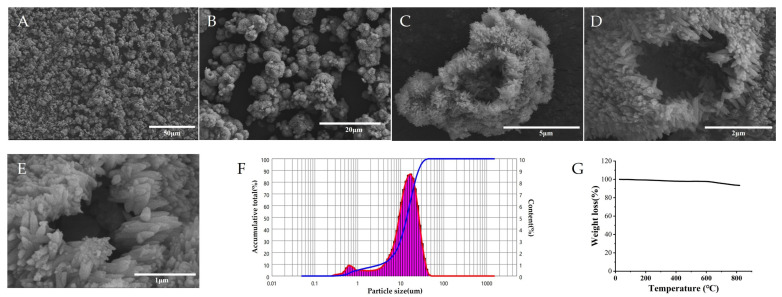
Physical characteristics of HAP. Note: (**A**–**E**) The morphology of HAP. (**F**) The result of the LDPSA. (**G**) The result of the TGA.

Figure 3A,B show that numerous HAP particles aggregated to form HAP clusters and these clusters distributed discretely. As shown in Figure 3C, the HAP particles exhibited an ellipsoidal morphology, and Figure 3D,E shows that the surface of HAP presented villous structures. In addition, each particle possessed a mass of tiny pores, and cavity structures existed in the particles. The results obtained with the LDPSA indicate that the particle sizes of the HAP particles ranged between 500 nm and 75 µm (Figure 3F) and the average particle size was 14.49 µm. The results of the mercury injection apparatus show that the total pore area of the HAP particles was 9.83 m^2^/g, the average pore diameter was 502.40 nm, the bulk density was 0.4415 g/mL, the apparent density was 0.9705 g/mL, and the porosity was 54.51%. The result of the TGA (Figure 3G) indicate that HAP possessed favorable thermal stability until 600 °C and it disintegrated when the temperature exceeded 600 °C.

### 3.4. Evaluation of PsP-HAP

#### 3.4.1. PsP-Loading Capacity and Cumulative Release Rate of PsP-HAP Sustained-Release System

**Figure 4:**
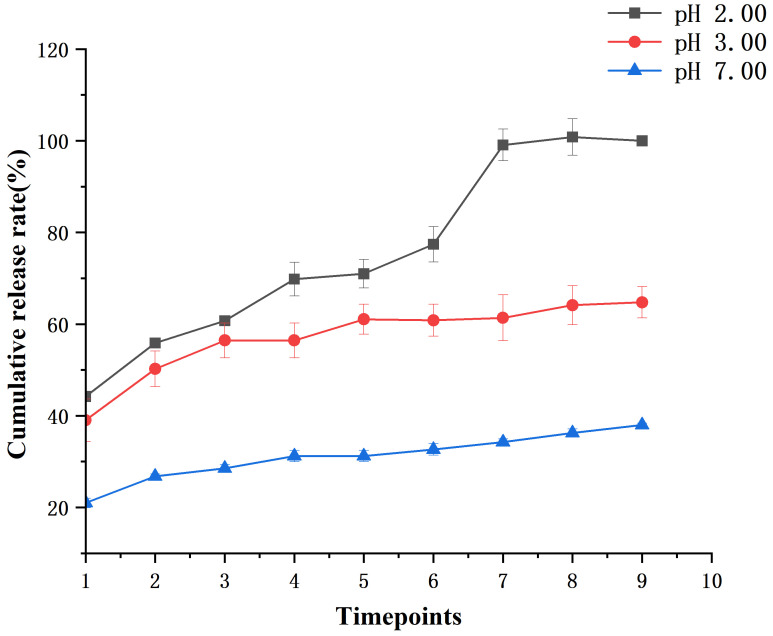
Cumulative release rates of PsP-HAP sustained-release system at pH 2.00, 3.00, and 7.00.

The results of the muffle furnace experiment indicated that the PsP content was 8.98% in PsP-HAP. Figure 4 shows the cumulative release rates of PsP-HAP at pH 2.00, 3.00, and 7.00. Within the 1.00st hour, they reached 44.22% (at pH 2.00), 39.05% (at pH 3.00), and 20.98% (at pH 7.00). Then, PsP-HAP’s cumulative release rates continuously increased before the 12.00th hour and tended to stability after that at the sustained-release condition of pH 2.00. At the sustained-release condition of pH 3.00, the cumulative release rates of PsP-HAP continuously increased before the 5.00th hour and tended to stability after that. However, the cumulative release rates of PsP-HAP only showed a small increase before the 2.00nd hour; it basically tended to stability after that at the sustained-release condition of pH 7.00. Finally, the cumulative release rates at pH 2.00, 3.00, and 7.00 reached 100.00%, 64.75%, and 38.00%, respectively, at the 48.00th hour. The above results indicate that PsP-HAP can have different sustained-release effects under different pH conditions. Therefore, it can be confirmed that PsP-HAP possesses a pH-responsive property, which can be used to realize controllable sustained-release effect to meet different physiological and health requirements.

#### 3.4.2. DPPH Radical Scavenging Rate of PsP-HAP Sustained-Release System

The measured results of the PsP concentrations in the sustained-release solutions of PsP-HAP at the five timepoints were 11.04 µg/mL, 2.63 µg/mL, 1.30 µg/mL, 0.21 µg/mL, and 0.14 µg/mL, respectively, and the antioxidative effect of the PsP-HAP group gradually decreased with the increase in time. The results related to the above two aspects indicate that the PsP concentrations in the sustained-release solutions of PsP-HAP were consistent with antioxidative activity.

**Figure 5:**
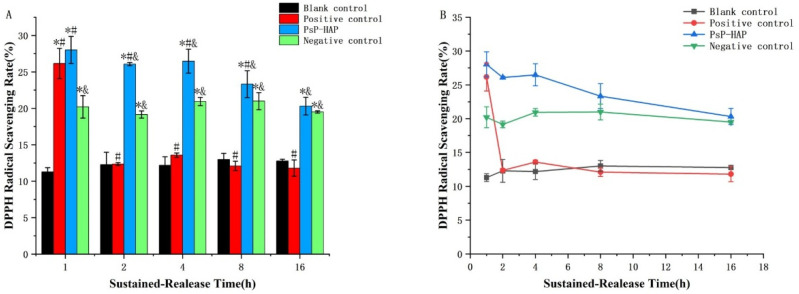
DPPH radical scavenging rate of PsP-HAP sustained-release system. Note: (**A**) Comparison of DPPH radical scavenging rate among different groups at each timepoint. (**B**) Comparison of DPPH radical scavenging rate among different timepoints in each group. *: The difference compared with the blank control group was statistically significant [11]. #: The difference compared with the negative control group was statistically significant [11]. &: The difference compared with the positive control group was statistically significant [11].

Figure 5B shows the DPPH radical scavenging rate of different solutions. The DPPH radical scavenging rate of the blank control group did not induce changes among the five timepoints (*p* > 0.05). The DPPH radical scavenging rate of the positive control group was significantly higher than that of the blank control group at the first timepoint, which indicates that PsP had a DPPH radical scavenging effect at the first timepoint (first timepoint: *F* = 64.890, *p* < 0.05); however, the difference did not exist at other timepoints, and there were no significant differences among the last four timepoints (*p* > 0.05). In addition, the negative control group also did not show significant changes in DPPH radical scavenging effect among the five timepoints (*p* > 0.05).

Regarding the antioxidative effect of the PsP-HAP sustained-release system, Figure 5B shows that there were no significant differences among the first timepoint, second timepoint, and third timepoint (*p* > 0.05). Figure 5A shows that the DPPH radical scavenging rate of the PsP-HAP sustained-release system was significantly higher than that of the blank control group at each timepoint (first timepoint: *F* = 64.890; second timepoint: *F* = 161.737; third timepoint: *F* = 119.518; fourth timepoint: *F* = 65.714; fifth timepoint: *F* = 84.486, *p* < 0.05) and was significantly higher than that of the negative control group, except for the last timepoint (first timepoint: *F* = 64.890; second timepoint: *F* = 161.737; third timepoint: *F* = 119.518; fourth timepoint: *F* = 65.714, *p* < 0.05). In addition, the DPPH radical scavenging rate of the PsP-HAP sustained-release system was significantly higher than that of positive control group, except for the first timepoint (second timepoint: *F* = 161.737; third timepoint: *F* = 119.518; fourth timepoint: *F* = 65.714; fifth timepoint: *F* = 84.486, *p* < 0.05).

#### 3.4.3. Cytotoxicity of PsP-HAP Sustained-Release System

**Figure 6:**
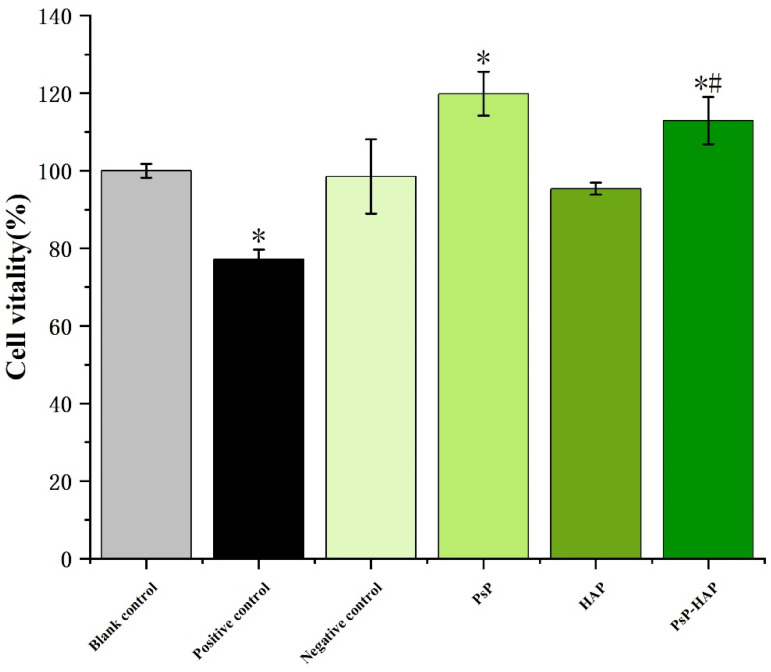
Cytotoxicity evaluation of PsP-HAP sustained-release system. Note: *: The difference compared with the blank control group was statistically significant. #: The difference compared with the HAP group was statistically significant.

Figure 6 shows that the cell proliferation vitality of the positive control group was significantly lower than the blank control group (*F* = 28.668, *p* < 0.05), indicating that the C2C12 cell stain can really be damaged by the toxicant. In addition, there were no significant differences between the HAP solution and the blank control group, indicating that HAP is relatively safe. The proliferative activities of PsP and PsP-HAP were significantly higher than that of the blank group (*F* = 28.668, *p* < 0.05), and PsP-HAP’s proliferative activity was significantly higher than that of HAP at the same time, indicating that PsP-HAP did not induce cytotoxicity. Finally, there were no significant differences in cytotoxicity between PsP-HAP and PsP (*p* > 0.05).

#### 3.4.4. Total Antioxidative Capacity of PsP-HAP Sustained-Release System

The measured results of the PsP concentrations in the sustained-release solutions of PsP-HAP at four timepoints were 11.04 µg/mL, 1.30 µg/mL, 0.21 µg/mL, and 0.14 µg/mL, and the antioxidative effect of the PsP-HAP group gradually decreased with the increase in time. The results related to the above two aspects indicate that the PsP concentrations in the sustained-release solutions of PsP-HAP were consistent with antioxidative activity.

**Figure 7:**
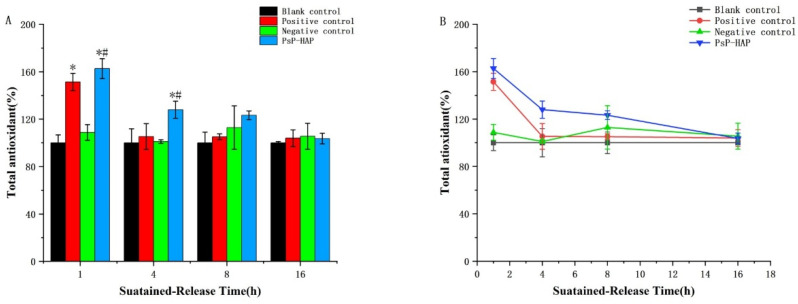
Total antioxidative capacity of PsP-HAP sustained-release system. Note: (**A**) Comparison of total antioxidative capacity among different groups at each timepoint. (**B**) Comparison of total antioxidative capacity among different timepoints in each group. *: The difference compared with the blank control group was statistically significant [11]. #: The difference compared with the negative control group was statistically significant [11].

Figure 7B shows the FRAP values of different solutions. The FRAP value of the blank control group was stable among 4 timepoints (*p* > 0.05). The FRAP value of the positive control group was significantly higher than that of the blank control group at the first timepoint, which indicates that PsP had total antioxidative capacity at this time (first timepoint: *F* = 36.268, *p* < 0.05); however, the difference did not exist at other timepoints (*p* > 0.05), and there were no significant differences among the last three timepoints (*p* > 0.05). In addition, the negative control group also appeared to have a stable trend among all timepoints, and there were no significant differences in FRAP value between the negative control group and the blank control group (*p* > 0.05).

Regarding the total antioxidative capacity of the PsP-HAP sustained-release system, Figure 7B shows that there were no significant differences between the second timepoint and the third timepoint (*p* > 0.05). Figure 7A shows that it was significantly higher than that of the blank control group and the negative control group at the first timepoint and the second timepoint (first timepoint: *F* = 36.268; second timepoint: *F* = 3.846, *p* < 0.05), respectively. In addition, it was also significantly higher than that of the positive control group at the second timepoint (second timepoint: *F* = 3.846, *p* < 0.05).

## 4. Discussion

In order to achieve full utilization of PsP, this study synthesized a new food formulation of PsP, a sustained-release system for PsP; optimized its key technical parameters by using the RSM; and evaluated its loading capacity, cumulative release rate, cytotoxicity, and antioxidative effect. As the result of RSM experiments, the optimal stirrer temperature, reaction pH, etching pH, and loading time were 85.62 °C, pH 11.12, pH 8.40 and 5.10 h, respectively. The evaluation indicators included two aspects, main sustained-release performance and in vitro functional effect of the sustained-release system, which indicated that the optimal PsP-HAP sustained-release system possessed sustained-release ability that could realize continuous release of PsP and can continuously exert an antioxidative effect.

The synthetic method of HAP included the template method and the precipitation method, which possess various advantages. The template method has better loading capacity as it can produce bigger hollow-core structures and more binding sites, and the precipitation method’s operation is simple, easy, and convenient [26,27]. At present, we do not find any research team who have used the template method to load polysaccharide. Because we need to obtain a greater loading amount of PsP so as to achieve a better sustained-release effect, this research study attempted to load this polysaccharide by using the template method. Our results show that the template method achieved a better loading effect (8.98%), which was close to the 13.60% of a similar study that used the precipitation method to load *Bletilla striata* Polysaccharide (BsP) into HAP [25]. The reason of the difference in the loading effect between the two kinds of methods might be the different molecular weight of PsP and BsP. We will conduct further studies about this difference in the next stage, and our team will continuously adopt the template method to load polysaccharide in the subsequent animal experiment.

The reason why the RSM design did not use CaCO_3_ dosage was that the single-factor experiments did not show the highest point under the condition where HAP purity was ensured. Some research studies [28,29] suggested that synthetic HAP is the purest when the ratio of Ca to P is 1.67:1; therefore, we explored encapsulation at different ratios by fixing the amount of Na_2_HPO_4_ and changed the CaCO_3_ dosage. As per our original scheme, optimal encapsulation should be achieved with 2.00 g of CaCO_3_ with the best ratio of Ca/P. However, the encapsulation did not show a significant decrease with more than 2.00 g of CaCO_3_, which might be because phosphate-deficient HAP under excessive CaCO_3_ can also load PsP. Therefore, CaCO_3_ dosage should not be used in the RSM design in subsequent research.

The single-factor experiments showed that pH 7.00 was the optimal etching pH, which is different from the condition (pH 6.00) of some references [30,31]. The reason is that their substance to be delivered might be more suitable for HAP with a more hollow-core structure that can be formed under acidic conditions. However, PsP might be more suitable for HAP with both a more hollow-core structure and more binding sites, which can be formed in a neutral environment but not under acidic conditions, because it is possible that high hydrogen-ion concentration can eliminate the binding sites of HAP. Then, the RSM experimental results further proved the above analysis, which indicated that pH 8.40 was the optimal etching pH parameter and also showed a possibility that an alkalescent condition can provide more binding sites for HAP to load PsP. Therefore, we adopted pH 8.40 as the optimal etching pH in the subsequent synthetic process of PsP-HAP.

The results of the physicochemical characterization of HAP indicate that the morphology, particle size, porosity, and thermal stability of the HAP that was synthesized by our study were basically the same as those in other similar studies [32,,33,34,35].

As we know, an acidic environment can degrade polysaccharide [36], and succus gastricus is just an acidic liquid [37,38]. Therefore, it is difficult to fully utilize the entire structure of PsP in the human body because it might be degraded in succus gastricus. The results of our preliminary experiment in C57BL6/J mice also indicated the same phenomenon. In order to reduce the degradation of PsP, stably maintain the content of the entire structure of PsP, and achieve fully utilization of the entire structure of PsP, we adopted HAP to load PsP, so as to obtain a new sustained-release food formulation for PsP, a PsP-HAP sustained-release system. Then, we used cumulative release rates of in vitro experiments to evaluate the effect of the PsP-HAP sustained-release system at different pH values which simulated the conditions of the digestive tract. The results indicated that 22.00–55.80% of PsP still existed in the PsP-HAP system within the first 6.00 h at pH 2.00 and 39.16–60.95% of PsP still existed in the PsP-HAP system within the first 6.00 h at pH 3.00; this provided two indirect pieces of evidence: the first one was that the PsP-HAP system can save PsP content, and the second one was that it is possible to reduce the adverse effect of succus gastricus on PsP to a certain extent. In addition, the above results also show that a change of one unit in acidic pH caused the release to be increased. The main reason is that HAP is a pH-responsive carrier [39,40]. It has been demonstrated that a mass of oxhydryl (OH^−^ group) on the surface of HAP can react with hydrions in an acidic environment, which leads to the dissolution of HAP [41], and the dissolution of HAP causes the release of PsP from PsP-HAP. It is obvious that there is a positive correlation between the concentration of hydrions and the release of PsP from PsP-HAP. So, the cumulative release rate increased from 64.75% to 100% when the pH changed from pH 3.00 to pH 2.00 in our research study. On the other hand, the PsP-HAP system showed continuous and slow release at the pH 7.00 level, which is an approximate pH environment (neural environment) [38,42] of the small intestine, within 48.00 h (48.00 h is the approximately digestion and metabolism time of the human body [43,44,45]), which indicates the possibility that 20.00–38.00% of PsP can exist whole in the small intestine [43]. The above in vitro experiments that simulated the conditions of the digestive tract proved that the PsP-HAP system can achieve a sustained-PsP-release effect, which provides the possibility to fully utilize the entire structure of PsP. It also provides reference to a certain extent for our animal experiment in the next stage.

The results of the cytotoxicity evaluation indicate that HAP was safe, which is in line with other studies [25,46,47]. Therefore, HAP can be used as a safe auxiliary material in producing food, and we can adopt HAP to load PsP without worrying about its cytotoxicity. These results again prove PsP-HAP’s usability from another perspective.

We did not find similar, previously published research studies where in vitro experiments on DPPH radical scavenging and cytological FRAP were adopted to prove the continuously active effect of substance delivery. In order to observe whether PsP-HAP really possesses a continuous antioxidative effect that is similar to PsP before animal experiments, we performed in vitro DPPH radical scavenging experiments and cytological FRAP experiments to further evaluate the effect and value of the PsP-HAP sustained-release system. In order to simulate the duration of digestion and absorption in the human body [43], we selected a time frame of 16.00 h to complete the two experiments. Both experiments showed that the PsP from the PsP-HAP sustained-release system still possessed the same antioxidative activity as free PsP at 1 h, which was significantly higher than that of the negative control and the blank control. After that, the result of in vitro DPPH radical scavenging indicated that the PsP from the PsP-HAP sustained-release system could still continuously produce a significant antioxidative effect at 8.00 h, which was significantly higher than that of the other three controls. The result of the cytological FRAP experiments indicate a similar antioxidative effect at 4.00 h, which was significantly higher than that of the other three controls. The two experiments proved the same fact from two angles; i.e., HAP did not reduce the effect and value of PsP. The reason might be that HAP does not destroy the original PsP structure in the process of loading PsP, and this will be explored by our team in the next stage. On the other hand, the two experiments also proved that the PsP-HAP sustained-release system not only can sustainably release PsP but also has long-lasting health-promoting properties on account of the consistent antioxidative effect exerted by whole PsP released from the PsP-HAP sustained-release system.

This research study synthesized a new food formulation for PsP, a PsP-HAP sustained-release system, and optimized and confirmed the optimal conditions under which HAP loads PsP. Then, it further evaluated the sustained-release ability of the PsP-HAP sustained-release system and the bioactive ability of PsP from the PsP-HAP sustained-release system, which can provide useful reference for efficiently utilizing PsP. Although the above results have achieved the research study’s target to a certain extent, we still need to perform animal experiments to further test these findings in the next stage of our research. In addition, we have noticed that some researchers [48,49,50,51] have used polysaccharides to load small bioactive molecule substances for enhancing their stability and bioavailability, so it would be interesting to explore another new food formulation that adopts PsP-HAP to load a small bioactive compound with cooperative synergic effect in further research. It is possible to conduct research of this kind on PsP in the future.

## 5. Conclusions

The physical characteristics of HAP can meet the requirements for loading PsP. A new food formulation of PsP, a PsP-HAP sustained-release system, has been constructed and optimized; it can achieve the largest PsP-loading effect and can achieve continuous release of PsP. At the same time, the continuous antioxidative effect of the PsP-HAP sustained-release system was firstly proved by the DPPH assay and cytologically by a total antioxidative capacity assay, which indicated that the PsP from PsP-HAP not only possessed the same antioxidative ability as free PsP at 1 h but can also continuously implement an antioxidative effect for 4–8 h. PsP-HAP can maintain the maximum content of PsP. This study successfully developed a new food formulation for PsP which shows the potential to stably maintain the content of PsP, achieving full utilization of PsP.

## Figures and Tables

**Table 1:** Reagents used in this research study.

Name of Product	Product Code	Company (City, State, Country)
Na_2_HPO_4_	D7292	Beijing Solarbio Science & Technology Co., Ltd. (Beijing, China)
CaCO_3_	239232	Sigma Co., Ltd. (Louis, MO, USA)
NaOH	240515D1	Xilong Scientific Co., Ltd. (Shantou, China)
Anthrone	A140968	Shanghai Aladdin Biochemical Technology Co., Ltd. (Shanghai, China) [10,11]
Phosphate buffer solution (PBS)	P1020	Beijing Solarbio Science & Technology Co., Ltd. (Beijing, China) [10,11]
DMEM culture medium	SH30243.01	Thermo Fisher Scientific, Inc. (Waltham, MA, USA) [10,11]
Fetal bovine serum	10091148	Thermo Fisher Scientific, Inc. (Waltham, MA, USA) [10,11]
*Polygonatum sibiricum* polysaccharide	2025060503	Shanxi Nanba Biotechnology Co., Ltd. (Taiyuan, China)
CCK-8 cell activity detection kit	C0037	Beyotime, Inc. (Shanghai, China) [10,11]
Total Antioxidant Capacity Assay Kit with FRAP method	S0116	Beyotime, Inc. (Shanghai, China) [10,11]
BCA Protein Assay Kit	P0012	Beyotime, Inc. (Shanghai, China) [10,11]
Citrate	C108869	Shanghai Aladdin Biochemical Technology Co., Ltd. (Shanghai, China)
DPPH	Y0303	Hefei BASF Biotechnology Co., Ltd. (Hefei, China) [10,11]

**Table 2:** RSM factors and levels for optimizing the synthetic parameters.

Levels	Factors			
	(A) Stirrer Temperature (°C)	(B) Reaction pH	(C) Etching pH	(D) PsP-Loading Time (h)
−1	60.00	10.60	5.00	3.00
0	85.00	11.30	7.00	6.00
1	110.00	12.00	9.00	9.00

**Table 3:** ANOVA of RSM for PsP-HAP.

Source	Sum of Squares	Df	Mean Square	F-Value	*p*-Value	
Model	1608.38	14	114.88	35.49	<0.0001	significant
A—Stirrer temperature	0.8216	1	0.8216	0.2538	0.6222	
B—Reaction pH	28.55	1	28.55	8.82	0.0101	
C—Etching pH	455.96	1	455.96	140.87	<0.0001	
D—PsP-loading time	5.63	1	5.63	1.74	0.2084	
AB	5.62	1	5.62	1.74	0.2089	
AC	2.79	1	2.79	0.8616	0.3690	
AD	4.45	1	4.45	1.38	0.2604	
BC	13.47	1	13.47	4.16	0.0607	
BD	32.66	1	32.66	10.09	0.0067	
CD	0.2070	1	0.2070	0.0640	0.8040	
A^2^	374.02	1	374.02	115.55	<0.0001	
B^2^	210.54	1	210.54	65.05	<0.0001	
C^2^	699.86	1	699.86	216.22	<0.0001	
D^2^	301.79	1	301.79	93.24	<0.0001	
Residual	45.32	14	3.24			
Lack of fit	41.54	10	4.15	4.40	0.0830	not significant
Pure error	3.78	4	0.9438			
Cor total	1653.69	28				

## Data Availability

The original contributions presented in this study are included in the article/Supplementary Materials. Further inquiries can be directed to the corresponding author.

## Supplementary Materials

The following supporting information can be downloaded at: https://www.mdpi.com/article/10.3390/foods15010147/s1, Table S1. RSM design’s experiment schedule and results.

## Abbreviations

PsP	*Polygonatum sibiricum* polysaccharide
HAP	Hydroxyapatite
PsP-HAP	Sustained-release system of *Polygonatum sibiricum* polysaccharide
RSM	Response surface method
DPPH	2,2-Diphenyl-1-picrylhydrazyl
FRAP	Ferric reducing antioxidant power
UAE-DES	Ultrasound-assisted extraction with deep eutectic solvents
PLGA	Poly(lactic-co-glycolic acid)
PBS	Phosphate buffer solution
DMSO	Dimethyl sulfoxide
BCA	Bicinchoninic acid
