# Supplementary material for: Synthesis, Optimization, and Evaluation of a New Sustained-Release Food Formulation for Polygonatum sibiricum Polysaccharide
Source: Foods. 2026 Jan 2;15(1):147. doi: 10.3390/foods15010147 (PMC12785529; doi:10.3390/foods15010147)
Supplement: Supplementary file 1 [file foods-15-00147-s001.zip › foods-3997576-supplementary.pdf]

Supplementary Materials:

Table S1. RSM design's experiment schedule and results.

	**Stirrer temperature (℃)**	**Reaction pH**	**Etching pH**	**PsP-loading time (h)**	**Encapsulation rate (%)**
1	60.00	10.60	7.00	6.00	28.21
2	110.00	10.60	7.00	6.00	28.08
3	60.00	12..00	7.00	6.00	20.03
4	110.00	12.00	7.00	6.00	24.64
5	85.00	11.30	5.00	3.00	13.61
6	85.00	11.30	9.00	3.00	27.40
7	85.00	11.30	5.00	9.00	15.70
8	85.00	11.30	9.00	9.00	28.58
9	60.00	11.30	7.00	3.00	22.48
10	110.00	11.30	7.00	3.00	24.85
11	60.00	11.30	7.00	9.00	26.14
12	110.00	11.30	7.00	9.00	24.29
13	85.00	10.60	5.00	6.00	15.62
14	85.00	12.00	5.00	6.00	15.45
15	85.00	10.60	9.00	6.00	33.76
16	85.00	12.00	9.00	6.00	26.25
17	60.00	11.30	5.00	6.00	15.87
18	110.00	11.30	5.00	6.00	16.61
19	60.00	11.30	9.00	6.00	26.72
20	110.00	11.30	9.00	6.00	24.12
21	85.00	10.60	7.00	3.00	28.49
22	85.00	12.00	7.00	3.00	23.17
23	85.00	10.60	7.00	9.00	23.7
24	85.00	12.00	7.00	9.00	29.81
25	85.00	11.30	7.00	6.00	37.52
26	85.00	11.30	7.00	6.00	38.14
27	85.00	11.30	7.00	6.00	39.85
28	85.00	11.30	7.00	6.00	39.56
29	85.00	11.30	7.00	6.00	38.59
